# Supplementary material for: Few-cycle laser driven reaction nanoscopy on aerosolized silica nanoparticles
Source: Nat Commun. 2019 Oct 11;10:4655. doi: 10.1038/s41467-019-12580-0 (PMC6789024; doi:10.1038/s41467-019-12580-0)
Supplement: Supplementary file 1 — Supplementary Information [file 41467_2019_12580_MOESM1_ESM.pdf]

Supplementary Information to

“Few-cycle laser driven reaction nanoscopy on aerosolized silica nanoparticles”

Rupp et al.

SUPPLEMENTARY NOTE 1

In order to directly visualize the three-dimensional proton momentum distribution $\rho(\theta,\varphi)$ (Main Fig. 3), we project it radially onto a unit sphere (Supplementary Fig. 1). The number dN of protons detected per solid angle $\mathrm{d}\Omega = \cos\theta\,\mathrm{d}\varphi\,\mathrm{d}\theta$ on this unit sphere is encoded in the color map of the surface plot:

$$\rho(\theta,\varphi) = \frac{\mathrm{d}N(\theta,\varphi)}{\mathrm{d}\Omega} = \frac{\mathrm{d}N(\theta,\varphi)}{\cos\theta\,\mathrm{d}\varphi\,\mathrm{d}\theta}$$

The azimuthal angle φ and the elevation angle θ are introduced to allow a convenient description in spherical coordinates. In that representation, the plotted proton yield is integrated over the kinetic energy. The spherical coordinates are defined as follows:

$$\begin{pmatrix}\text{propagation axis}\\ \text{jet axis}\\ \text{polarization axis}\end{pmatrix} = \begin{pmatrix} r\ \cos\theta\cos\varphi \\ r\ \cos\theta\sin\varphi \\ r\ \sin\theta\end{pmatrix}$$

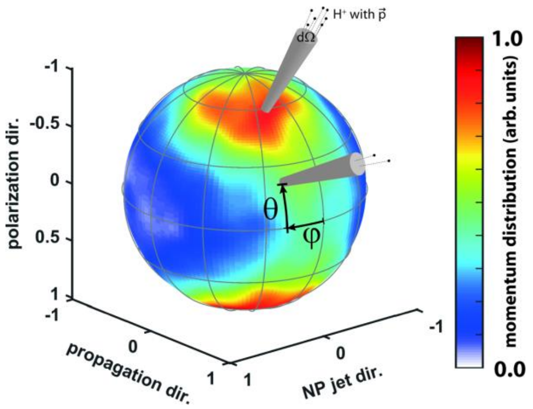

Supplementary Figure 1. Projection method. The 3D proton momentum distribution measured in the reaction nanoscope is projected radially onto a unit sphere. The proton density is encoded in the color map (Fig. 3c).

SUPPLEMENTARY NOTE 2

In order to estimate the effect of a possible relaxation of the surface charge distribution into an isotropic charge distribution during the propagation of the protons, we replace, after a certain time delay, the initial surface charge distribution on the nanoparticle by an isotropic distribution with the same total charge. The calculation is repeated for time different delays (0 ps, 1 ps, and 5 ps) after the interaction with the laser pulse (see Supplementary Figs. 2 and 3). After 5 ps, the protons reach a distance from the surface that is on the order of one nanoparticle diameter. While the case of an

immediate relaxation corresponds to the identity map of the initial angular position coordinate and final angular momentum coordinate, a later relaxation results in a slight blurring of the final momentum distribution. As can be seen in Supplementary Figs. 2 and 3, the effect of possible charge relaxation dynamics on the final momentum distribution is negligible for the conditions of our experiment

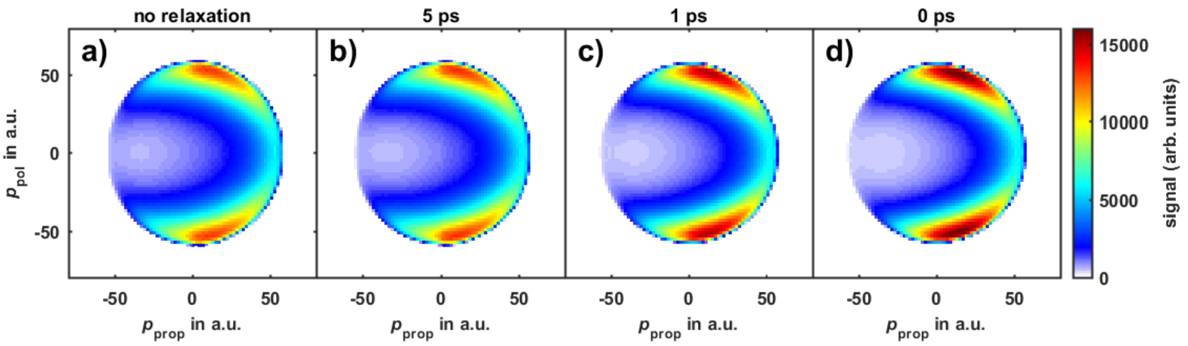

Supplementary Figure 2. Effect of relaxation processes. The final proton momentum distribution is calculated for different relaxation times of the surface charges distribution. (a) No relaxation is equivalent to the simulation in Fig. 6. (b-d) The surface charge distribution relaxes into an isotropic distribution after 5 ps, 1 ps and 0 ps the interaction with the laser pulse.

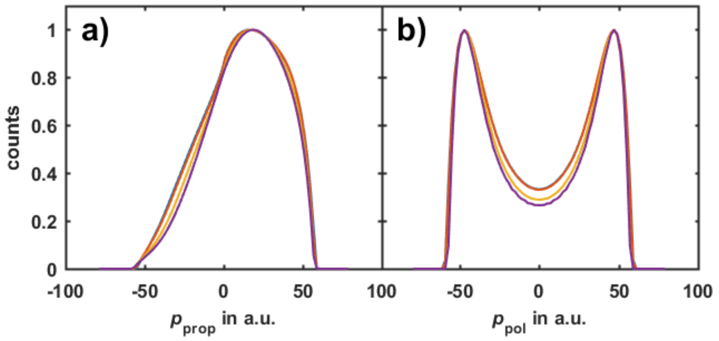

Supplementary Figure 3. Projected momentum distributions. Projection of the momentum distribution shown in Supplementary Fig. 2 onto different coordinate axis: propagation axis (a) and polarization axis (b). The different colors refer to the time at which relaxation occurs.

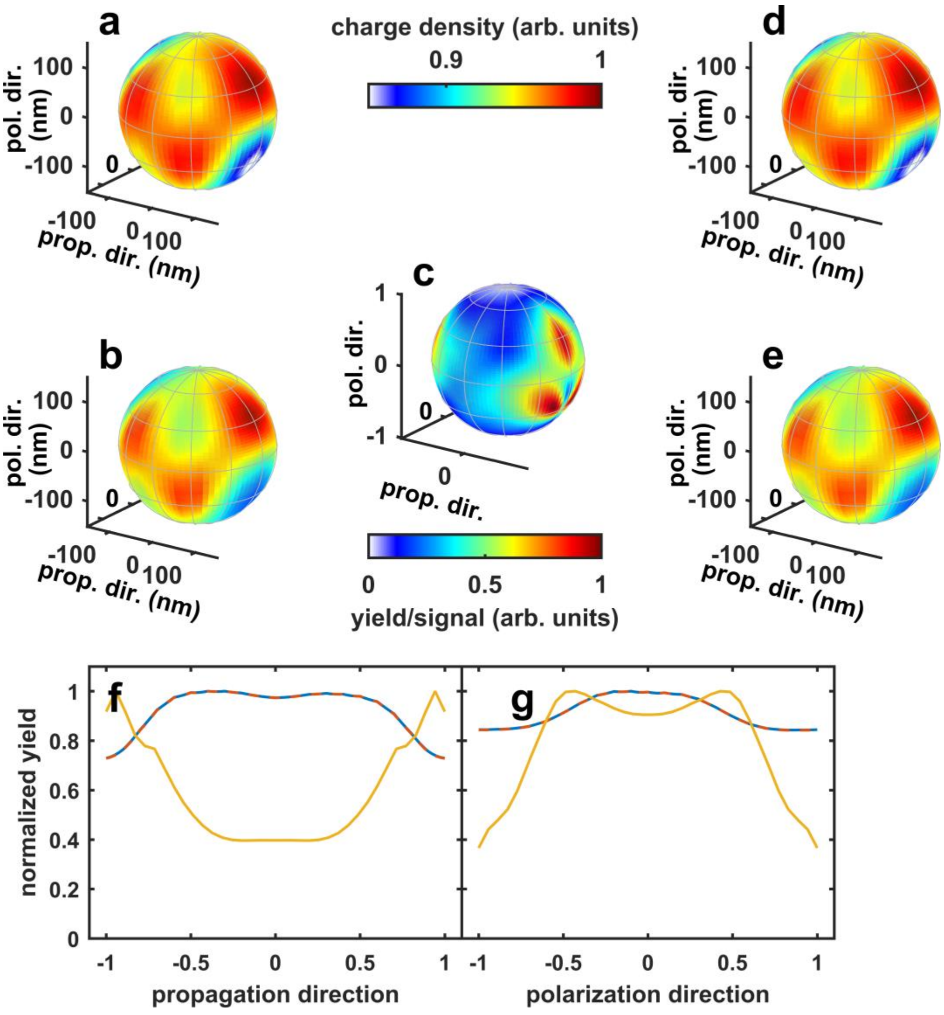

Supplementary Figure 4. Retrieval algorithm for complex dissociation yield distributions on a 100 nm nanoparticle. a) Surface charge distribution, b) surface yield distribution, c) final momentum distribution, d) retrieved surface charge distribution, e) retrieved surface yield. f, g) Initial (blue) and retrieved (red) surface yield distribution on the surface of the nanoparticle projected on the propagation (f) and polarization (g) axes. The yellow line in (e, f) is the projection of the final proton momentum distribution onto the propagation and polarization axis, respectively.

SUPPLEMENTARY NOTE 3

A detailed description of the Mean field Mie Monte-Carlo (M^3C) calculations is given in Supplementary Refs. [1, 2, 3] including the mathematical formulas describing the fields and the equations of motion. Below we summarize the main aspects of the M^3C simulations:

(1) The linear-response contribution to the near-fields is obtained via a spectral decomposition of the incident laser pulses in combination with (frequency-dependent) spatial modes for the spherical geometry, that are calculated analytically utilizing the Mie solution of Maxwell's equations. The obtained spatially enhanced near-fields (see Supplementary Fig. 5) provide a proper description of the nanoparticle's electromagnetic response to the incident pulses for moderate intensities, at which the local polarization of bound electrons (dielectric medium) is mostly linear with respect to the applied electric fields. We therefore denoted this field as the linear near-field in the context of this work. We like to note that the Kerr-type non-linear bound state polarization is much smaller than the linear contribution in the considered intensity range. Possible deviations in the near-field enhancement due to the Kerr-effect are estimated to be well below 5% for the considered experimental conditions.

(2) With increasing intensity, nonlinear response effects resulting from free carriers from ionization become increasingly important. The displacement of the free electrons from their host ions gives rise to an additional polarization that can exceed the bound state polarization and must be included (plasma response). We include both, the polarization of the sphere resulting from the free charges as well as the Coulomb interaction among the free charges, via a quasistatic, self-consistent mean field that we evaluate by solving Poisson's equation (using high order multipole expansion). A formal derivation of the underlying splitting of the fields into the linear term and the correction term including the non-linear response is given in [3].As the liberated electrons are not removed from the model for the bound state polarization in point (1) above (this would break the linearity needed for the spectral decomposition), the relative ionization per atom should remain small. This is fulfilled in the scenarios treated in our study.

(3) At every time step, tunneling ionization is evaluated by Monte-Carlo methods and considering an atomic tunneling rate (ADK-rate) for the local intensity resulting from the combined linear near-field and the mean field. Upon successful ionization events electron trajectories are launched and propagated by integration of classical equations of motion in the combined near-fields.

The self-consisted treatment of the dynamics in the model stages (1)-(3) form the employed M^3C description.

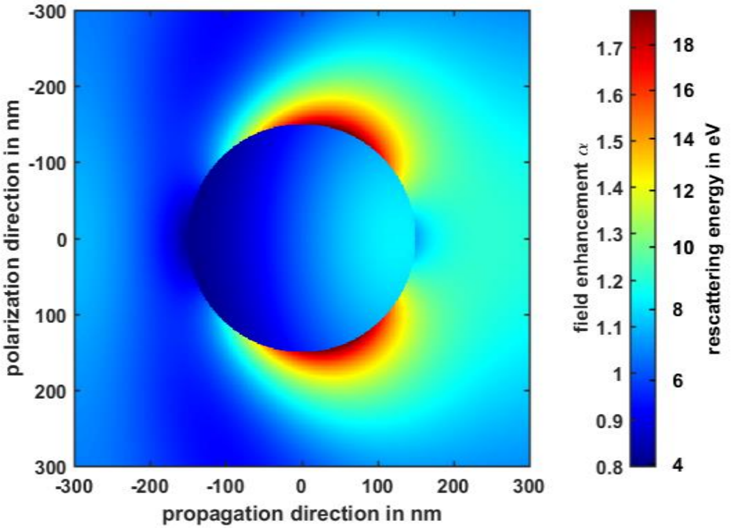

Supplementary Figure 5: Near-field distribution around the nanoparticle. The plot shows the field distribution around a 300 nm SiO_2 particle for a 720 nm wavelength laser field with an intensity of 4×10^{13} W cm^{-2}. The color denotes the field enhancement α relative to the incident field strength E_0 and the associated rescattering energy ($3.2\times\alpha^2\times U_p$). The highest field enhancement α is ~1.8 for the Mie solution. Including the self-consistent mean field (not shown, see Supplementary Ref. [4]) the maximum total field is enhanced to about 2.3 relative to E_0.

Supplementary References:

1. Süßmann F., *et al.* Field propagation-induced directionality of carrier-envelope phase-controlled photoemission from nanospheres. *Nat. Commun.* **6**, 7944 (2015).

2. Seiffert L., *et al.* Competition of single and double rescattering in the strong-field photoemission from dielectric nanospheres. *Appl. Phys. B* **122**, 1–9 (2016).

3. Seiffert L. Semi-classical description of near-field driven attosecond photoemission from nanostructures. PhD thesis, University of Rostock (2018).

4. Seiffert L., *et al.* Trapping field assisted backscattering in the strong-field photoemission from dielectric nanospheres. *J. Mod. Opt.* **64**, 1-8 (2016).
